# Report on the 3rd Board Meeting of the International Human Phenome Consortium

**DOI:** 10.1007/s43657-022-00065-y

**Published:** 2022-06-22

**Authors:** Mei Tian, Han Liu, Shunling Chen, Zhong Yang, Weishuo Tao, Shiwen Peng, Huiting Che, Li Jin, The Secretariat of International Human Phenome Consortium

**Affiliations:** 1 Human Phenome Institute, Fudan University, Shanghai, 200438 China; 2 International Human Phenome Institutes (Shanghai), Shanghai, 200433 China; 3 School of Life Sciences, Fudan University, Shanghai, 200438 China; 4 Shanghai Medical College, Fudan University, Shanghai, 200032 China

## Introduction

The phenome is a set of measurable traits, including the physical, chemical and biological traits of individuals and populations, that result from the complex interactions of genes, epigenetics, symbiotic microorganisms, diet and environmental exposures (Jin 2021). The aim of human phenomics is to precisely measure the human phenotypes and to comprehensively analyze the human phenome. In doing so, we can systemically deconstruct the relationship among perceived phenotypes, compile phenotypic network, expand multi-dimensional and cross-scale correlations between macro and micro phenotypes, and eventually clarify the correlations among these phenotypes. After the completion of the Human Genome Project, there is an urgent need for discovering the multi-level association among genes, environment and phenotypes to complement the other half of the required information for human health. Therefore, Fudan University has initiated the International Human Phenome Project in 2018, providing a new opportunity for biomedical research and leading the development of biomedicine.

The International Human Phenome Consortium (IHPC) was co-founded by Dr. Jeremy Nicholson from Murdoch University (Australia), Dr. Leroy Hood from the Institute for Systems Biology (United States), and Dr. Li Jin from Fudan University (China) in 2018. The Board of IHPC, consisting of 24 prestigious scholars from 20 countries, serves as a steering committee to guide the strategy and implementation of the International Human Phenome Project and to ensure coordination across the IHPC members. The IHPC has three sub-committees. These committees include the Technology and Standard Committee, the Ethics, Law and Societal Issues Committee and the Data Sharing Committee, all devoted to establishing harmonized protocols for standardized phenotyping measurement, data processing, practical issues and shared informatics. The consortium aims to build the leading institutions to form a global innovation network dedicated to large-scale research efforts on human phenome, promoting human phenome projects globally. The international network of these integrated efforts is designated to explore the correlations among genes, environment and phenotypes at the microscopic or macroscopic levels, as well as their corresponding mechanisms. The mission of IHPC is to utilize such a network to discover new information and models to understand the origin and diversity of human traits or diseases from the molecular, cellular to organismal level. Through the application of a diverse range of physical, chemical and biological metrics, as well as novel discoveries and technologies, IHPC intends to create novel paradigms for curing diseases and improving human health.

**Fig. 1:**
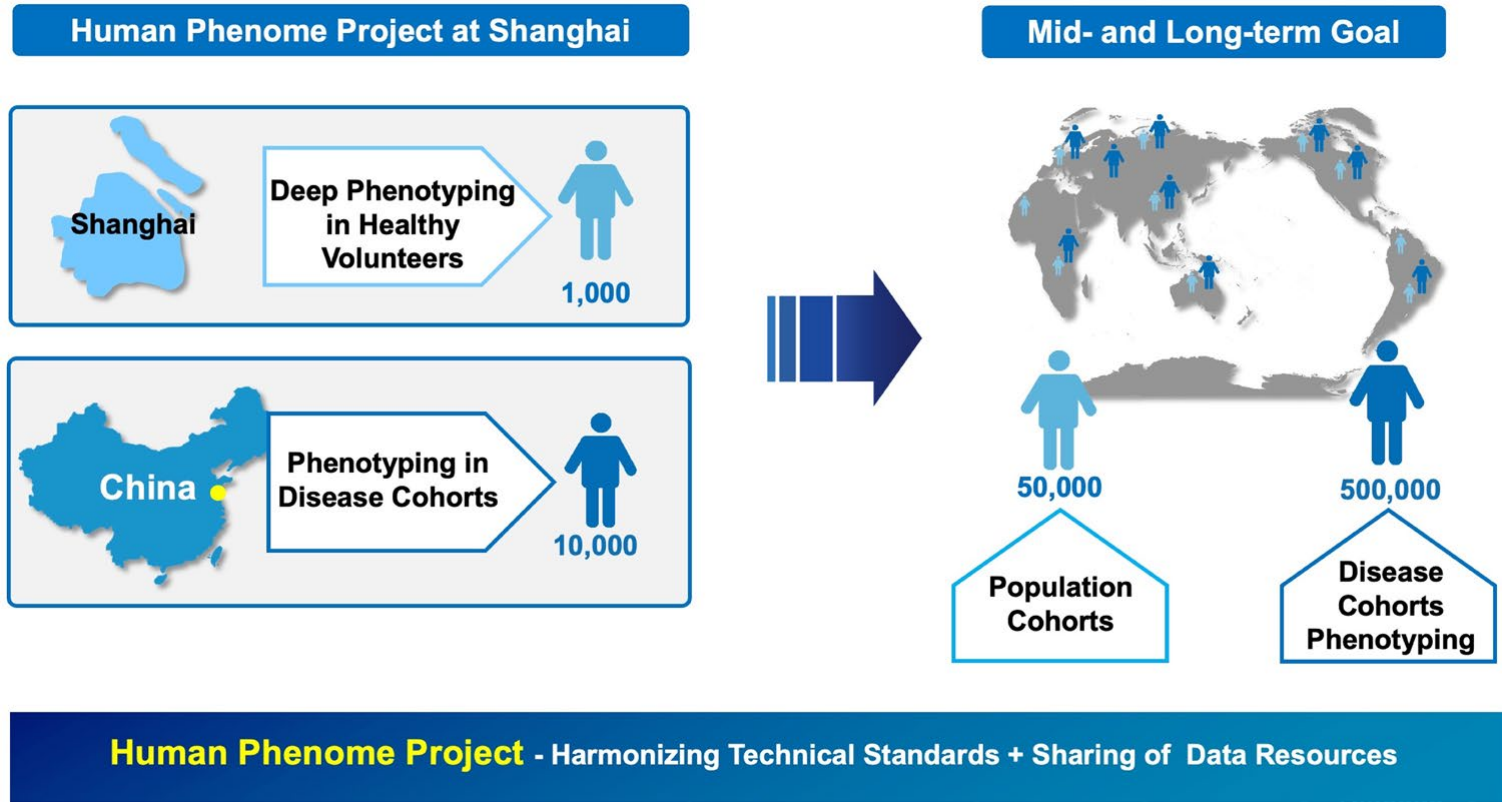
Overview of Human Phenome Project

The International Symposium of Human Phenomics is the most important frontier academic summit in the field of phenomics and has been held by IHPC twice to date. At the International Symposium of Human Phenomics in 2018, the IHPC specified the implementation road map, cooperation mechanism and organizational structure of the International Human Phenome Project (Fig. 1). At the International Symposium of Human Phenomics in 2020, the members of the IHPC reached an important consensus on the three near-term priorities of the human phenome project, including: (1) phenomics of new coronary pneumonia and other major diseases; (2) technology system and research infrastructure for phenome research; and (3) Standard Operating Procedures (SOPs) in phenome studies. In addition, the consortium formally announced the establishment of Secretariat of IHPC at the International Human Phenome Institutes (Shanghai), Shanghai, China.

The 3rd IHPC Board Meeting was held virtually on November 19th, 2021, and 30 outstanding scientists from 18 countries, including board members, new nominees and invited guest scholars, attended the meeting. The meeting was convened by the Secretariat of IHPC and co-hosted by the International Human Phenome Institutes (Shanghai) and the Human Phenome Institute of Fudan University.

## Keynote Lectures

The keynote lectures were delivered by the three co-founders of the IHPC, Dr. Li Jin, Dr. Jeremy Nicholson, and Dr. Leroy Hood. These lectures introduced the latest progress in human phenome studies, including the world’s first human phenome navigation atlas, COVID-19 molecular phenomics, and the Beyond the Human Genome (BHG) project.

## I: Human Phenome Navigation Atlas

Dr. Li Jin, principal of Fudan University and the co-founder of the IHPC, gave a talk on his exploratory research of the world’s first human phenome navigation atlas.

To identify the relationships among human phenotypes, the Human Phenome Institute of Fudan University and the International Human Phenome Institutes (Shanghai) have together established cross-scale and multi-dimensional human phenotyping platforms for deep phenotyping covering over 30,000 phenotypes in 15 categories. This provides a one-stop solution to the measurement of human phenotypes at both macro- and micro-levels.

**Fig. 2:**
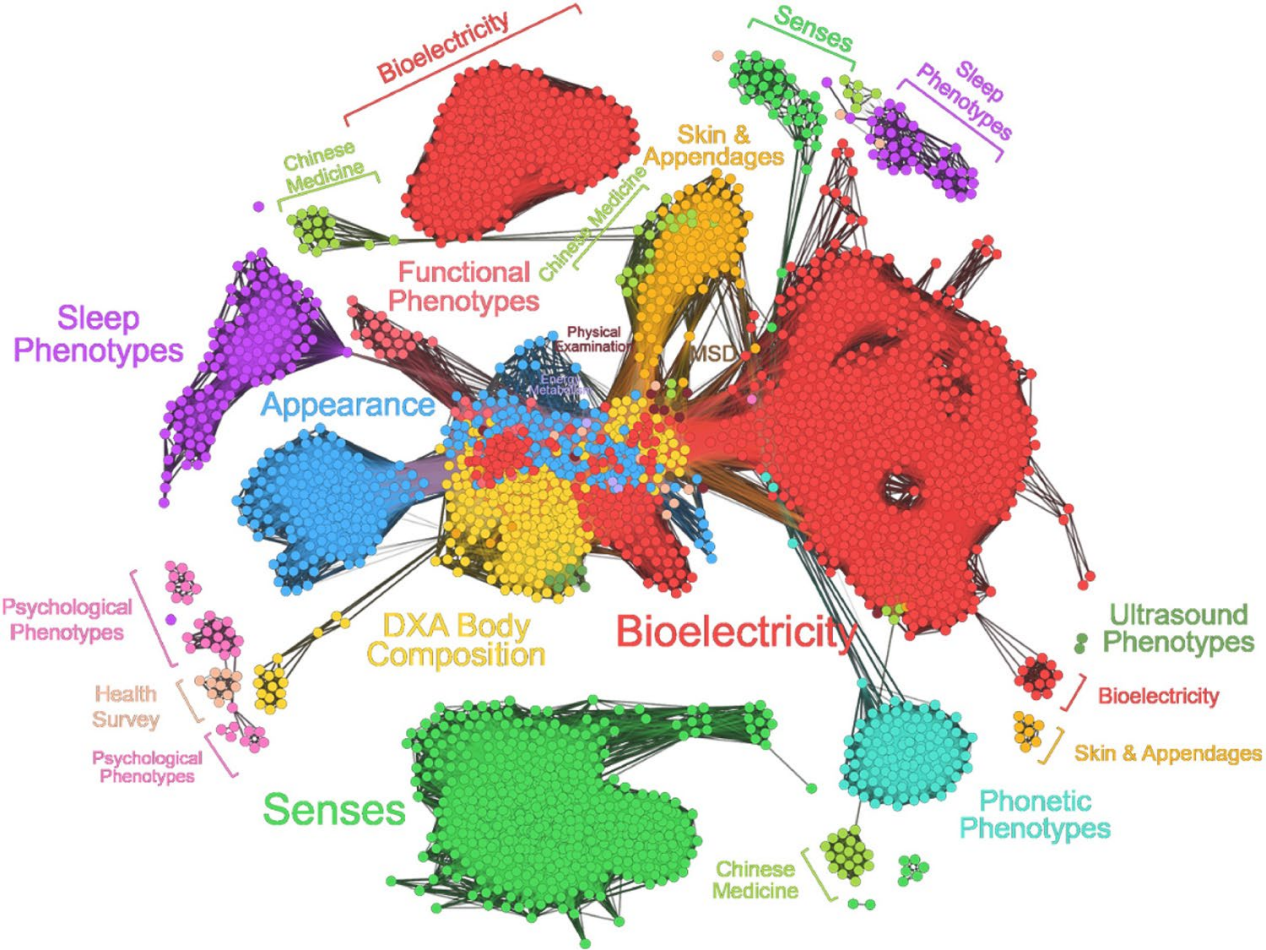
The Human Phenome Navigation Atlas

Currently, the team has established a core cohort to conduct a longitudinal study on phenotypes of healthy people. Over 740 healthy volunteers have participated in the study, and 30,974 phenotypes have been collected from each individual. Consequently, 1.5 million connections with strong statistical significance among phenotypes were identified, which formed a human phenome navigation “atlas” with a huge phenotypic correlation network (Fig. 2). The atlas is essential for scientific exploration and decoding of the life sciences that will eventually facilitate precision healthcare management.

## II: COVID-19 and Molecular Phenomics

Research on molecular phenomics can provide essential chemical and biochemical information (proteins, transcripts, metabolites, etc.) to understand emergent diseases and their systemic effects. Metabolic phenotyping measures the compositions of metabolites in biofluids and tissues, which provides physiological or pathological status of an individual and the metabolic trajectory in the disease process (Nicholson et al. 2012).

Dr. Jeremy Nicholson, director of the Australian National Phenome Center (ANPC) and co-founder of the IHPC, gave a keynote presentation with an overview on how to define the natural history of COVID-19 with molecular phenomics. As SARS-CoV-2 is spreading worldwide, the ANPC has shifted its research focus to COVID-19. Novel NMR spectroscopic and mass spectrometric technologies are the most readily applied methods to generate multivariate profiles of metabolites for exploratory and targeted analysis. These advanced metabolic phenotyping methods and comprehensive data analyses have provided deep insight into biomarker signatures and metabolic pathways of the disease, such as lipoproteins (Kimhofer et al. 2020; Lodge et al. 2021a, 2021c; Loo et al. 2020), glycoproteins (Lodge et al. 2021b), lipids (Lawler et al. 2021) and other key metabolites (Gray et al. 2021), as well as the correlations between these metabolic parameters and inflammatory cytokines/chemokines (Bergamaschi et al. 2021; Lawler et al. 2021).

Based on the concept of phenoconversion, a process in which an individual moves from a healthy state to a disordered pathophysiological state, the research team has proposed a phenoconversion model for the detection and prevention of diseases in terms of metabolic profiles (Kimhofer et al. 2020). This process is associated with many biomarkers demonstrating disease severity and status. Phenoreversion, the process of recovery from SARS-CoV-2 infections, is the opposite of disease phenoconversion. However, due to the complex systematic effects of COVID-19, many patients exhibit persistent symptoms and multi-organ damage associated with the post-acute COVID-19 Syndrome (PACS) (Holmes et al. 2021). The recovery process could be highly variable among patients, and one cannot be considered as “fully recovered” until the biochemical profile returns to normal. The predictive, diagnostic and prognostic metabolic phenotyping information allows clinical actionability by understanding the pathways perturbed by the COVID-19 disease.

## III: Beyond the Human Genome

Dr. Leroy Hood, the co-founder of the Institute for Systems Biology and the IHPC, introduced a one-million-people project recently initiated by himself, entitled “Beyond the Human Genome (BHG): inventing the Science of Wellness and Prevention”. The project is directed by a non-profit organization, Phenome Health (https://phenomehealth.org/), which was established in October 2021. The BHG project is proposed to carry out longitudinal phenome analysis for the body, brain, gut microbiome, and genome sequencing of one million individuals over ten years across the U.S. to convert these data into actionable possibilities and optimize health trajectory.

In 2017, Dr. Hood started a company, Arivale (https://keep.health/arivale/), which recruited 5,000 participants for deep genome and phenome analysis over four years. This population has provided proof of principle for precision population health and has led to the science of wellness and prevention. The project's vision is to acknowledge that everyone has their own trajectory consisting of three components: wellness, the transition from wellness to disease, and disease progression up to clinical signs. The transition from wellness to disease often occurs many years before the first clinical symptoms appear. Therefore, the main objective of the BHG project is to be able to detect the early transition markers and reverse all transitions of diseases prior to clinical manifesting to wellness.

This critical scientific project also facilitates the implementation of “P4 medicine”, which refers to predictive, preventive, personalized and participatory (Hood et al. 2012). This concept has been advocated by Dr. Hood and his research team for years and was considered the largest paradigm shift in the history of medicine. Among the four “Ps”, the participation of patients and physicians is believed to be the most challenging aspect. Collaborating with different partners allows the Phenome Health team to access the electronic records of patients and creates opportunities to seek powerful new solutions to conquer the foremost striking challenges of contemporary healthcare: quality costs, rapid population expansion, aging population and the explosion of chronic diseases.

## Reports

Dr. Akihiro Umezawa from National Center for Child Health and Development (Japan) and Dr. Mitali Mukerji from the Indian Institute of Technology Jodhpur (India) presented their recent research progress and innovative projects in the report session.

## I: iPS (Induced Pluripotent Stem) Cell-Organoids for Genetic Disorder and Related Ethical Issues

On behalf of the Committee on Ethics, Law, and Societal Issues, Dr. Akihiro Umezawa (National Center for Child Health and Development, NCCHD, Japan) focused his presentation on the use of iPS cell-organoids to treat genetic disorders and its related ethical issues. The NCCHD has established the use of iPS cells for rare genetic diseases and applied organoid induction technology to elucidate the etiology and pathogenesis of these diseases. In addition, iPS cell research is also the hotspot of ethical and societal issues. Dr. Umezawa emphasized that it is important to receive informed consent from patients before utilizing these iPS cells for research and commercial purposes. He then introduced the two acts that govern cell therapy and regenerative medicine in Japan. Commercial regenerative and cellular therapeutical products that seek market approval need to follow the *Pharmaceuticals and Medical Device Act* (PMD Act), while early small-scale clinical research conducted by physicians in the hospital involving processed cells should comply with the *Act on the Safety of Regenerative Medicine* (RM Act) (Tobita et al. 2016).

## II: “Thar DESIGNS” Project

Dr. Mitali Mukerji (Indian Institute of Technology Jodhpur, India) introduced the “Thar DESIGNS (Desert EcoSystem Innovations Guided by Nature & Selection)” project, which is an initiative on ecosystem phenomics. Despite the abundance of minerals and carbon sinks, the Thar desert has a hostile living environment that challenges all living creatures in the ecosystem. The extreme conditions have given rise to unique phenotypes in terms of cellular, physiological, and anatomical behaviors. The project is dedicated to capturing phenome and genome data from the local ecosystem to the regional level, and to understanding the physiological and molecular basis of the signatures of adaption using advanced technologies. The project is intended to provide a “Desert Ecosystem Knowledge Grid” that could foster the cycle of engineering-research-development-commercialization, which could facilitate the development of actionable interventional strategies for common and endemic diseases in the desert regions, as well as the promotion of ecological conservation and restoration.

## Discussion

Dr. Jean Krutmann (Leibniz Research Institute of Environmental Medicine, Germany), the co-chair of the Committee on Data Sharing, presented a proposal entitled “Principles of Trans-Border Data Sharing and Openness of the Human Phenome Project”. The sharing and openness of global scientific research data across borders is a *conditio sine qua non* of the scientific research of human phenome. The International Human Phenome Project attaches great importance to trans-border data sharing and openness. Under the spirit of upholding security and openness, the IHPC board members have passed the proposal unanimously during the discussion session.

## Future Perspectives

The meeting presented cutting-edge progress in the Human Phenome Project. It is encouraging that there have been many scientific research achievements under the framework of the International Human Phenome Project, such as breakthroughs in molecular phenomics, early cancer screening, multi-omic biomarker identification. Most importantly, the first preliminary Human Phenome Navigation Atlas was made public at the meeting. Through deep conversations among board members and invited scholars, all participants have reached a consensus on the Principles of Trans-Border Data Sharing and Openness of Human Phenome Project, which provides a basis for the International Human Phenome Project research teams around the world to measure and share data under unified standards in the future.

The IHPC will continue working on international cooperation and scientific and technological exchanges of human phenomics. We hope that the development of human phenomics can accelerate the leap of human precision measurement technology and standards, and finally achieve the goal of serving human health. More outstanding experts and scholars are welcome to join the IHPC.

## Data Availability

Not applicable.
